# Antenatal care utilization and nutrition counseling are strongly associated with infant and young child feeding knowledge among rural/semi-urban women in Harari region, Eastern Ethiopia

**DOI:** 10.3389/fped.2022.1013051

**Published:** 2022-09-29

**Authors:** Aklilu Abrham Roba, Assefa Tola, Desta Dugassa, Maleda Tefera, Tadese Gure, Teshager Worku, Awugchew Teshome Ayele, Tamirat Tesfaye Dasa, Hailemariam Mekonnen Workie, Melese Mengistu Asfaw, Abiyot Asfaw, Firehiwot Mesfin, Lemma Demissie Regassa, Yadeta Dessie, Fitsum Abera, Meron Yeshitila, Meskerem Girma, Muluken Gezahagn, Feven Tezera, Nega Assefa, Kedir Teji Roba

**Affiliations:** ^1^College of Health and Medical Sciences, Haramaya University, Dire Dawa, Ethiopia; ^2^Haramaya Institute of Technology, Haramaya University, Dire Dawa, Ethiopia; ^3^College of Medicine and Health Sciences, Arba Minch University, Arba Minch, Ethiopia; ^4^College of Health Science, Hawassa University, Hawassa, Ethiopia; ^5^College of Medicine and Health Sciences, Bahir Dar University, Bahir Dar, Ethiopia; ^6^College of Agriculture and Natural Resource, Wolkite University, Wolkite, Ethiopia; ^7^College of Agriculture, Haramaya University, Dire Dawa, Ethiopia

**Keywords:** infant and young child feeding, antenatal follow up, nutrition counseling, knowledge, pregnant women

## Abstract

There is a gap in evidence linking antenatal care (ANC) utilization, nutrition counseling, and knowledge of pregnant women about infant and young child feeding (IYCF), particularly in low-income settings. Therefore, this study aimed to identify the association between ANC follow-up and nutrition counseling with IYCF knowledge. A cross-sectional study was conducted among 390 pregnant women in the rural kebeles of the Harari region from January to June 2019. Data were collected using face-to-face interviews on tablet computers. Bivariate and multivariate logistic regression were employed. An adjusted odds ratio (with 95% CI) was used to determine the strength of association between IYCF knowledge with ANC follow-up and nutrition counseling by adjusting for educational status, occupation, gravida, and distance to the nearest health center. Overall, 54.4% [95% CI 49.2, 59.2] of currently pregnant women were knowledgeable about IYCF of which only 20% started ANC follow-up and 24.4% received nutrition counseling. Out of 288 multigravida women, only 51.4% had ANC follow-up during their last pregnancy. In the adjusted model, ANC follow-up during the current pregnancy (AOR 1.85, 95% CI 1.07–3.22), those who received nutrition counseling (AOR 1.92, 95% CI 1.09–3.38), literate in education (AOR 1.71, 95% CI 1.07–2.73), multigravida (AOR 1.96, 95% CI 1.12–3.43), and far from the nearest health center (AOR 0.95, 95% CI 0.93–0.97) were significantly associated with the mothers IYCF knowledge. Thus, health care providers should encourage mothers to attend ANC during pregnancy and provide nutrition counseling about the IYCF.

## Introduction

Early life nutrition, particularly during the first 1,000 days from conception to end of the 2nd year of a child, has an important influence on the growth and development of the child. This period is the “critical window”, the time in which the infant is the most vulnerable and the time when crucial brain development occurs (1). A child needs the right kind of nutrition at the right time to thrive and achieve optimal growth and development. Appropriate nutrition during this period forms an important foundation for optimal growth, health, and brain development for the future life of a child (1–4). The most appropriate feeding strategy to ensure optimal feeding for an infant and young child is the initiation of breastfeeding within 1 h of birth, keeping exclusive breastfeeding for the first 6 months, and continue breastfeeding for 2 years or beyond, along with sufficient, safe, and appropriate complementary feeding around 6 months (1, 4, 5).

Undernourished children face learning difficulties in school, earn less in later life, and face barriers to participate in their communities (4). The burden of undernutrition among under-five children remains unacceptably high throughout the world (6). Childhood undernutrition is a major public health problem in developing countries that adversely affect individuals, families, and the community at large (4). In Ethiopia, it is a deep-rooted public health problem among under-five children with stunting (37%), underweight (21%), and wasting (7%). Moreover, among these children, 12% were severely stunted, 6% were severely underweight, and 1% were severely wasted (7).

Undernutrition in children results from multiple factors (8). Maternal knowledge of recommended child feeding practices like exclusive breastfeeding for 6 months, continued breastfeeding, and timely transition to adequate complementary feeding is a key factor for optimal nutritional status, health, and growth of the children (9–13). Lack of knowledge about IYCF is the leading cause of undernutrition among infants and young children in many developing countries (14). Undernutrition is not always only the result of a lack of food but is associated with a lack of knowledge about optimal feeding practices and the provision of poor-quality food (13). The IYCF knowledge of pregnant women is crucial for promoting optimal breastfeeding practices and general child feeding practices in the first 1,000 days of life (2, 15–17).

Antenatal care (ANC] is a key strategy to improve nutrition and health knowledge and promote preventive health practices in low-resource settings (18). ANC service is an ideal entry point to provide multiple health and nutritional interventions to promote maternal and fetal wellbeing, breastfeeding behaviors, and birth preparedness. It presents a unique and lifesaving opportunity for health promotion, disease prevention, early diagnosis, and treatment of illnesses during pregnancy (19). Some studies demonstrated that ANC service utilization increases institutional delivery, utilization of postnatal care, and infant feeding behavior (20–22).

In Ethiopia, the trends of ANC utilization from a skilled provider have increased from 28% in 2005 to 74% in 2019. Also, 71% women of reproductive age received nutrition counseling during their ANC follow-up in 2019 (23). However, the effect of ANC follow-up on maternal IYCF knowledge is not yet adequately studied for evidence-based program planning and intervention in resource-limited rural settings. Thus, as part of a baseline study for a randomized controlled trial (Trial no.: PACTR201804003012418), the current study aimed to determine the association between ANC follow-up and nutrition counseling with IYCF knowledge among pregnant women in the rural areas of the Harari region, Eastern Ethiopia.

## Methods and materials

### Study area and period

This study was carried out in rural kebeles of Harari regional state, located 525 km east of Addis Ababa, the capital of Ethiopia. The region has nine woreda administrations (six urban and three rural) with 19 urban kebele administrations and 17 rural peasant associations. The region has a total of 2,40,000 people, of which 1,23,072 were females and 53,383 are women in the reproductive age group in 2016 (24). The region has an estimated population density of 589.05 people per square kilometer with 3.9 persons per household, 3.4 and 4.6 for urban and rural households, respectively. There are six hospitals, one regional laboratory and research center, eight health centers, 32 health posts, and 40 private clinics in the region (25). The study was conducted from January to June 2019.

### Design and population

A community-based cross-sectional study was carried out as part of a randomized controlled trial (RCT) entitled ‘Effect of Locally Formulated Complimentary Food and Enhanced Homestead Food Production Interventions on Maternal, Infant and Young Child Nutritional Status in the First 1,000 Days in Rural Kebeles of Harari Region, Eastern Ethiopia with trial registration PACTR201804003012418'. The first 1,000 days refers to a child's life from the day he/she is conceived to he/she has completed 2 years of age (24 months) (26). All amenorrheic mothers for at least 3 months were screened for pregnancy by Human Chorionic Gonadotrophin hormone (HCG urine test) by trained data collectors with the help of health extension workers in their respective households. For HCG confirmed women, ultrasound was used to confirm the pregnancy and estimate the gestational age. The study included pregnant women residing for more than 6 months in randomly selected rural kebeles.

### Sample size and sampling techniques

The sample size which was calculated for the RCT (Trial no.: PACTR201804003012418) was used for the current study with the following assumptions: 28% of Harar women meet adequate gestational weight gain (27), overall trial-wise Type I error rate of 0.05 (α *p* < 0.05); 80% power. By adding a 10% drop-out rate, the study recruited 100 women in each arm and obtained a total sample size of 400 pregnant women.

For the current study, a *post hoc* power analysis using G^*^Power 3.1.9.4 software was used to determine the power of the study (28). Based on the odds ratio between IYCF knowledge and ANC follow-up (1.85) and nutrition counseling (1.92), an alpha of 0.05, the sample size of 390, and a one-tailed distribution, a power of 0.99 was determined.

Pregnant women were selected using a two stages sampling procedure. First, from a total of 17 rural kebeles of the Harari region, eight were selected by simple random sampling. The randomly selected kebeles were Sukul, Hasen-Gay, Aboker Muti, Erer Ulanula, Dire Teyara, Sofi, Harewae, and Qile. Health extension workers visited every household to identify and recruit the study population in the selected kebeles. Pregnancy was asserted in three stages: mother's claim of her last menstrual period, urine test for pregnancy (HCG), and finally confirmed by ultrasound examination. The total sample size was distributed proportionally to the selected kebeles. When there was more than one pregnant woman in the household, one was selected by lottery method.

### Data collection and variables

The data were collected by four trained nurses (BSc. holders) using a pretested structured questionnaire. English version of the questionnaire was adapted from the Ethiopian Demographic and Health Survey (EDHS) questionnaires which is standardized version (29, 30). It was translated to local languages (Amharic and Oromiffa) and back-translated to English by individuals with good command of both languages (English and Amharic or English and Oromiffa). A pretesting of these questionnaires was done to ensure that the wordings were appropriate to yield the required data by 20 pregnant women in the nearby kebele. Identified gaps between the English and local language versions were corrected before the actual data collection. Data was collected by tablet using ODK open-source software to reduce data entry errors. The questionnaire contained sociodemographic characteristics of the woman and her partner, current and past obstetric history, IYCF knowledge, etc. The IYCF knowledge of the woman was generated using the mean scores based on eight knowledge questions. Respondents who scored below the mean were categorized as “poor knowledge” and vice versa.

The dependent variable was IYCF knowledge while the primary explanatory variable was ANC follow-up for the current pregnancy and nutrition counseling. In addition, the study considered the following as potential confounders: educational status, occupational status, gravida, ever used modern family planning, and distance to the nearest health center.

### Data analysis

The normality of data was determined using Shapiro-Wilk's test. Visual inspection of histograms and box plots were used to detect outliers. Percentages and mean (with standard deviation) were used to describe the findings. A binary logistic regression model was employed to identify the crude association between each variable with IYCF knowledge. All variables which had a *p*-value < 0.2 in the bivariate analysis were included in the multivariate binary logistic regression model. An adjusted odds ratio was used to determine the strength of the association between the dependent and independent variables. Statistical significance was determined using a *p*-value < 0.05. Analysis was conducted using Stata version 16.0 (31).

## Results

### Sociodemographic characteristics

A total of 390 pregnant women were included in this analysis with a response rate of 97.5%. The ages range from 15 to 44 years with a mean (±SD) of 25.6 and 5.8 years. All of them were married and Muslims in their religion. Around half (49.7%) of them had never attended formal education. The majority were housewives (91.5%) and their partners were farmers (62.3%). The mean (±SD) duration to reach the nearest health post, health center, and a hospital was 19.7 (±10.5), 24.5 (±12.4), and 51.4 (±13.5) minutes, respectively. Public transport was the main means of transportation (Table 1).

**Table 1:** Sociodemographic characteristics of currently pregnant women in rural areas of Harari region, eastern Ethiopia, 2019.

**Variables**		**Number**	**Percentage (%)**
Age	15–24 years	182	46.7%
	25–34 years	167	42.8%
	35–49 years	41	10.5%
Educational status	Illiterate	194	49.7%
	Grade 1–8	157	40.3%
	More than grade 8	39	10.0%
Occupation of pregnant women	Housewife	357	91.5%
	Business and others	33	8.5%
Occupation of partner	Farming	243	62.3%
	Business and others	147	37.7%
Means of transportation to the nearest health post	Walking by foot	159	40.8%
	Public transport	178	45.6%
	Private transport	53	13.6%
Means of transportation to nearest health centers	Walking by foot	25	6.4%
	*Public transport*	303	77.7%
	*Private transport*	62	15.9%

### Current obstetrics characteristics

The majority (80%) of currently pregnant women have not started antenatal care follow-up, while only half (48.5%) of the women had a history of ANC follow-up in the previous pregnancies. Similarly, 295 (75.6%) of currently pregnant women did not receive nutrition counseling about their children during their current and previous pregnancies. Only 42 (10.8%) of currently pregnant women had a history of using modern family planning methods before this pregnancy. The number of pregnancies ranges from 1 to 11 times with the mean (±SD) of 3.36 (2.3). Similarly, the mean (±SD) of live birth was 2.32 (2.28). On the other hand, the number of currently alive children ranges from 0 to 11 with an average of 2.27 children per woman (Table 2).

**Table 2:** Obstetric characteristics of currently pregnant women in rural areas of Harari region, eastern Ethiopia, 2019.

**Variables**		**Number**	**Percentage (%)**
Married	Within a year	74	19.0
	2–5 years	121	31.0
	6–10 years	101	25.9
	More than 10 years	94	24.1
Ever used modern FP	No	348	89.2
	Yes	42	10.8
FP use before this pregnancy (*n* = 42)	No	6	14.29
	Yes	36	85.71
Gravidity	Primigravida	102	26.2
	Multigravida	288	73.8
Parity	Nulliparous	103	26.4
	Primiparous	78	20.0
	Multiparous	209	53.6
Abortion history	No	371	95.1
	Yes	19	4.9
Stillbirth history	No	379	97.2
	Yes	11	2.8
ANC for current pregnancy	No	312	80.0
	Yes	78	20.0
TT vaccine	No	291	74.6
	Yes	99	25.4
Obtained nutrition counseling	No	295	75.6
	Yes	95	24.4

### Past obstetric history

Out of 288 multigravida women, 148 (51.4%) had ANC follow-up during their previous (last) pregnancy. Only 33 (22.3%) of them completed ANC follow three times and none of them attained it four times. Among the 99 women who have TT vaccination history, only 12 (12.1%) of them took it three times and the remaining took it twice (50.5%) or once (37.4%). Regarding the place of delivery of the last child, 141 (49%) mothers delivered in their own home (33.0%) or their mother's home (16%). The birth interval between the last child and the current pregnancy ranged from <1–10 years with two-thirds (63.9%) of mothers having 2–5 years intervals. The majority (82.6%) of mothers exposed their last child to sunlight. Among the index children (the last before the current one), only 58 (20.1%) and 103 (35%) took vitamin A supplements and deworming, respectively. Even if 248 (86.1%) of mothers responded as they knew the benefit of vaccination of their children, only 51 (17.7%) of children have been fully vaccinated. Furthermore, only half (49.7%) of the children had BCG scars (Table 3).

**Table 3:** Past obstetric history of currently pregnant women in rural areas of Harari region, eastern Ethiopia, 2019 (*n* = 288).

**Variable**		**Number**	**Percentage (%)**
ANC for the last pregnancy	No	140	48.6
	Yes	148	51.4
ANC follow up	Once	38	25.7
(*n* = 148)	Twice	77	52.0
	Three times	33	22.3
Place of birth for the last child	Own Home	95	33.0
	Mother's Home	46	16.0
	Governmental Hospital	79	27.4
	Health Center	67	23.3
	Private Clinic	1	0.3
Birth interval of your last 2 children	Less than 2 years	90	31.2
	2–5 years	184	63.9
	More than 5 years	14	4.9
Vitamin A supplement	No	230	79.9
	Yes	58	20.1
Exposed to Sunlight	No	50	17.4
	Yes	238	82.6
Child take deworming	No	185	64.2
	Yes	103	35.8
Know the benefits of child vaccination	No	40	13.9
	Yes	248	86.1
Child fully vaccinated	No	81	28.1
	Yes	51	17.7
	Has no card	156	54.2
BCG scar (observed)	No	145	50.3
	Yes	143	49.7

### IYCF knowledge of pregnant women

The majority (93.8%) of pregnant women heard about immediately giving breast milk to the baby after birth. The main sources of information for these women were family and friends (38.2%) and health extension workers (37.4%). Similarly, the majority (81.5%) of pregnant women knew the duration of exclusive breastfeeding. Only 60.5% of the pregnant women correctly mentioned the time to start complementary feeding. Overall, 54.4% [95% CI 49.2, 59.2] of currently pregnant women were knowledgeable about IYCF (Table 4).

**Table 4:** Knowledge about infant feeding among currently pregnant women in rural areas of Harari region, eastern Ethiopia, 2019.

**Variables**	**Number**	**Percentage**
Heard when to put the baby on the breast after birth	366	93.8
Information from health professionals	220	60.1
Know when to initiate breastfeeding for the newborn	287	73.6
Know the duration of exclusive breastfeeding	318	81.5
Know about colostrum	362	92.8
Know the importance of colostrum for the newborn	321	82.3
Know when to initiate complementary feeding	236	60.5
Know the duration of breastfeeding	247	63.3
**Overall knowledge**
Not knowledgeable	178	45.6
Knowledgeable	212	54.4

### Factors associated with IYCF knowledge

In the adjusted model, ANC follow-up in the current pregnancy, nutrition counseling, educational status, gravida, and distance from the nearest health center were significantly associated with the IYCF knowledge. Pregnant women who already initiated their ANC follow-up in the current pregnancy were 1.85 times (AOR = 1.85; 95% CI 1.07–3.22) more likely to have IYCF knowledge compared to those who didn't have ANC follow-up. Similarly, the odds of having IYCF knowledge among the women who received nutrition counseling were twice (AOR =1.92; 95% CI 1.09–3.38) better than their counterparts. Literate women were 1.71 times (AOR = 1.71; 95% CI 1.07–2.73) more likely to have better knowledge about IYCF than their illiterate counterparts. The probability of having IYCF knowledge among multigravida women was almost twice (AOR = 1.96; 95% CI 1.12–3.43) higher than primigravida women. Finally, when the distance from the nearest health center increased by a kilometer, there was a 5% decrease in knowledge about IYCF (AOR 0.95, 95% CI 0.93–0.97) (Table 5).

**Table 5:** Factors associated with Knowledge about infant feeding of currently pregnant women in rural areas of Harari region, eastern Ethiopia, 2019.

**Variables**	**Knowledge about infant feeding**		**COR (95% CI)**	**AOR**
	**Not knowledgeable**	**Knowledgeable**		**(95% CI)**
**Educational status**				
Illiterate	104	90	Reference	Reference
Literate	74	122	0.34 (0.16–0.72)	1.71 (1.07–2.73) *
**Occupational status**				
Housewife	166	191	Reference	Reference
Business and others	12	21	0.66 (0.31–1.38)	0.56 (0.25–1.26)
**Gravida**				
Primigravida	52	50	Reference	Reference
Multigravida	126	162	0.75 (0.48–1.18)	1.96 (1.12–3.43) *
**ANC for current pregnancy**				
No	130	182	Reference	Reference
Yes	48	30	2.24 (1.35–3.73)	1.85 (1.07–3.22) *
**Ever used modern FP**				
No	157	191	Reference	Reference
Yes	21	21	1.22 (0.64–2.31)	1.73 (0.85–3.52)
**Nutritional counseling**
No	136	159	Reference	Reference
Yes	42	53	0.93 (0.58–1.47)	1.92 (1.09–3.38) *
Distance/far from the nearest health center			0.95 (0.93–0.96)	0.95 (0.93–0.97) **

^*^Denotes p-value < 0.05,

^**^denotes p-value < 0.001.

## Discussion

The current study identified ANC follow-up in the current pregnancy, nutrition counseling, educational status, gravida, and distance from the nearest health center as predictors of the IYCF knowledge. Also, it revealed that 54.4% of currently pregnant women were knowledgeable about IYCF. This figure was consistent with previous studies including West Gojjam Zone of Northwest Ethiopia (60%) (9), Adea Woreda of the Oromia region in Ethiopia (51%) (32), Moshi Urban of Tanzania (61.2%) (15), and Kpandai district of the Northern Region of Ghana (50%) (12). However, the IYCF knowledge in this study was higher than the findings of the Cross River State of Nigeria (20%) (13), Sagamu of southwestern Nigeria (41.4%) (33), and Kenya (46%) (18). But it was less than studies conducted in Nnewi South-East Nigeria (82.0%) (34) and the Wolaita Zone of southern Ethiopia (66.1%) (35). This variation might be due to the difference in the study population, sample size, operational definition of knowledge, and socio-economic backgrounds of the participants (13, 33–35).

Regarding the specific knowledge levels, 81.5% of mothers knew the recommended duration of exclusive breastfeeding, which was consistent with the findings done in the Shabelle Zone of the Somali Region in Eastern Ethiopia that stated 85% of the mothers knew the correct time for exclusive breastfeeding (10). In this study, 73.6% of respondents correctly mentioned immediately initiating breastfeeding after delivery. It is consistent with the study conducted in Tanzania (71%) (15) but higher than studies conducted in Shabelle Zone of Somali region (47%) (10) and in China (32.5%) (36). Furthermore, 60.5% of the mothers correctly mentioned the right time to introduce complementary feeding is at completed 6 months which was consistent with a study done in rural Hebei Province of China (63.9%) (36) but lower than the result found in Moshi Urban of Tanzania (83%) (15). Moreover, in the current study, about two–thirds (63.3%) of pregnant women correctly mentioned the duration of breastfeeding after initiating additional food. This finding was much lower than a study conducted in the Shabelle Zone of the Somali Region in Eastern Ethiopia (91%) (10). Those variations might be due to differences in the study settings and the socioeconomic status of study participants.

Maternal educational status is one of the important determinants of IYCF knowledge (37). Educated mothers can better understand the information provided by health professionals regarding IYCF than illiterate women. The positive association between maternal educational status and their IYCF knowledge was reported by previous studies (9, 17, 33, 37). However, a study in the Shabelle Zone of the Somali Region in Eastern Ethiopia presented maternal educational status did not show any significant association with the IYCF knowledge (10).

The number of lifetime pregnancies (Gravida) was also associated with the IYCF knowledge. Accordingly, those multigravida pregnant women were more knowledgeable about IYCF than primigravida. Consistent findings were reported from other similar studies as pregnant women with a higher number of children had more knowledge than those with few children (17, 35). A study in Arba Minch Zuria of southern Ethiopia showed that mothers with <3 children had less chance of being knowledgeable about optimal child feeding practices (17).

Besides improving maternal and child outcomes, attending ANC has the potential to increase maternal knowledge about dietary diversity for self and IYCF (18, 38). Our study also showed that those pregnant women who started ANC follow-up in their current pregnancy were more knowledgeable about IYCF than those who did not start ANC follow-up. It agrees with studies conducted in the Wolaita Zone of Southern Ethiopia (35), West Gojjam Zone of Northwest Ethiopia (9), Arba Minch Zuria of southern Ethiopia (17), and Western Kenya (18), and Sagamu of southwestern Nigeria (33). ANC attending is an excellent opportunity to receive health-related information. This information can improve the knowledge of attendants.

Although ANC is a good contact point to get pregnant mothers and educate them about IYCF, only few women received nutrition information (36). Our study also revealed that those pregnant women who received nutrition counseling during current and previous pregnancies had better knowledge than those who did not receive it. Similar findings were reported in previous studies (9, 15, 35). A study by Hashim et al. stated that those women who received counseling on breastfeeding during the current pregnancy had almost 4 times higher knowledge about appropriate breastfeeding knowledge (15).

Strengths of this study include pregnancy was confirmed objectively by HCG urine test and ultrasound. Also, the data was collected by tablet computers to reduce errors during data collection and entry. Furthermore, as this study is part of a randomized controlled trial, due emphasis was given to data quality from designing to the analysis stage. Though there are several strengths, it has some limitations. The study included pregnant women in rural/semi-urban areas of eastern Ethiopia. Thus, the findings may not reflect their urban counterparts. Finally, since the data was collected at a single point, a temporal relationship may not be established.

## Conclusion and recommendations

The overall IYCF knowledge of pregnant women was fair and associated with ANC follow-up, maternal education status, distance from the nearest health center, number of lifetime pregnancies (gravida), and nutrition counseling in current/previous pregnancies. Thus, improving the utilization of antenatal care and incorporating nutritional counseling is a crucial intervention to increase the IYCF knowledge, particularly in primigravida women. Local government administrators and other stakeholders should emphasize women's education, as educated women have a better consciousness of IYCF practices. The health care providers, mainly health extension workers, should encourage mothers to attend antenatal care during pregnancy and provide IYCF education.

## Data availability statement

The raw data supporting the conclusions of this article will be made available by the authors, without undue reservation.

## Ethics statement

The Ethical clearance was obtained from Haramaya University College of Health and Medical Sciences Institutional Health Research Ethics Review Committee (IHRERC/068/2018). The patients/participants provided their written informed consent to participate in this study.

## Author contributions

AAR and all the co-authors: conceived and designed the study, acquisition of the fund and data, and critical revision of the manuscript. AAR and DD: project and resource administration. AAR, DD, MT, ATA, TTD, HW, MA, and KTR: supervision. AAR: original draft. All authors contributed to the article and approved the submitted version.

## Funding

Haramaya University, Office of Research Affairs (HURG−2017-02-02-09).

## Conflict of interest

The authors declare that the research was conducted in the absence of any commercial or financial relationships that could be construed as a potential conflict of interest.

## Publisher's note

All claims expressed in this article are solely those of the authors and do not necessarily represent those of their affiliated organizations, or those of the publisher, the editors and the reviewers. Any product that may be evaluated in this article, or claim that may be made by its manufacturer, is not guaranteed or endorsed by the publisher.

## Abbreviations

ANC: antenatal care
AOR: adjusted odds ratio
CI: confidence interval
HCG: human chorionic gonadotropin
IYCF: infant and young child feeding
ODK: open data kit
RCT: randomized controlled trial
SD: standard deviation
TT: tetanus toxoid.
